# Annexin A1 Regulates NLRP3 Inflammasome Activation and Modifies Lipid Release Profile in Isolated Peritoneal Macrophages

**DOI:** 10.3390/cells9040926

**Published:** 2020-04-09

**Authors:** José Marcos Sanches, Laura Migliari Branco, Gustavo Henrique Bueno Duarte, Sonia Maria Oliani, Karina Ramalho Bortoluci, Vanessa Moreira, Cristiane Damas Gil

**Affiliations:** 1 Departamento de Morfologia e Genética, Universidade Federal de São Paulo, São Paulo 04023-900, Brazil; ms.marcossanches@gmail.com; 2 Faculdade de Medicina, Universidade do Oeste Paulista, Guarujá, São Paulo 11410-980, Brazil; 3 Departamento de Ciências Biológicas e Centro de Terapia Celular e Molecular, Universidade Federal de São Paulo, São Paulo 04044-010, Brazil; laurambranco@gmail.com (L.M.B.); karina.bortolucci@unifesp.br (K.R.B.); 4 Instituto de Química, Universidade Estadual de Campinas, Campinas, São Paulo 13083-862, Brazil; gustavo_duarte95@hotmail.com; 5 Programa de Pós-Graduação em Biociências, Instituto de Biociências, Letras e Ciências Exatas (IBILCE), Universidade Estadual Paulista, São José do Rio Preto, São Paulo 15054-000, Brazil; sonia.oliani@unesp.br; 6 Departamento de Farmacologia, Universidade Federal de São Paulo, São Paulo 04044-020, Brazil; vmoreira@unifesp.br

**Keywords:** inflammation, nigericin, pyroptosis, mass spectrometry, lipidomics

## Abstract

Annexin A1 (AnxA1) is a potent anti-inflammatory protein that downregulates proinflammatory cytokine release. This study evaluated the role of AnxA1 in the regulation of NLRP3 inflammasome activation and lipid release by starch-elicited murine peritoneal macrophages. C57bl/6 wild-type (WT) and AnxA1-null (AnxA1^-/-^) mice received an intraperitoneal injection of 1.5% starch solution for macrophage recruitment. NLRP3 was activated by priming cells with lipopolysaccharide for 3 h, followed by nigericin (1 h) or ATP (30 min) incubation. As expected, nigericin and ATP administration decreased elicited peritoneal macrophage viability and induced IL-1β release, more pronounced in the AnxA1^-/-^ cells than in the control peritoneal macrophages. In addition, nigericin-activated AnxA1^-/-^ macrophages showed increased levels of NLRP3, while points of co-localization of the AnxA1 protein and NLRP3 inflammasome were detected in WT cells, as demonstrated by ultrastructural analysis. The lipidomic analysis showed a pronounced release of prostaglandins in nigericin-stimulated WT peritoneal macrophages, while ceramides were detected in AnxA1^-/-^ cell supernatants. Different eicosanoid profiles were detected for both genotypes, and our results suggest that endogenous AnxA1 regulates the NLRP3-derived IL-1β and lipid mediator release in macrophages.

## 1. Introduction

Annexin A1 (AnxA1) is a 37-kDa protein that can mimic the anti-inflammatory action of glucocorticoids by inhibiting eicosanoid and phospholipase A2 synthesis, affecting components of inflammatory reaction and arachidonic acid release [1,2]. The ability of AnxA1 to down-modulate cellular and molecular processes of inflammation contributes to tissue homeostasis and reprogramming of macrophages [3]. The development of the AnxA1-null mice (AnxA1^-/-^) strain has allowed for a better understanding of the role of endogenous AnxA1 protein in leukocyte biology and the inflammatory process. In models of inflammation induced by carrageenan or zymosan, AnxA1^-/-^ animals exhibit an exacerbated response characterized by a prominent leukocyte influx and IL-1β release [4]. In addition, macrophages from AnxA1^-/-^ mice demonstrate reduced ability to phagocytose non-opsonized zymosan particles [5], and show higher TLR4 mRNA expression and IL-1β production after lipopolysaccharide (LPS) stimulation than wild-type cells [6]. These data demonstrate that AnxA1 exerts a negative inflammatory response through its down-modulation effects on macrophage cells, which are important leukocytes in an innate response. 

In addition, the stimulation of a P2 × 7 receptor (P2X7R) in resting and M2 macrophages, but not in M1 cells, provokes the rapid release of AnxA1 through its exposure with phosphatidylserine to the outer plasma membrane leaflet [7]. Also, the release of AnxA1 after P2X7R activation is not affected in inflammasome knockout macrophages, suggesting that its release is independent of caspase-1 activation. Considering that P2X7R activation is necessary to promote the assembly of the NLRP3 inflammasome and cytokine release [8], the release of AnxA1 represents another P2X7R macrophage signaling pathway for the resolution of the inflammation. 

Nucleotide-binding oligomerization domain (NOD)-like receptor family pyrin domain containing 3 (NLRP3 or NALP3) is a cytoplasmic sensor that oligomerizes to form a platform known as inflammasome, a protein complex that controls the release of IL-1β and IL-18 by activating caspase-1 [9]. In macrophages, NLRP3 inflammasome activation can be triggered by the pore-forming ionophore nigericin, extracellular ATP, and crystalline substances that induce pyroptosis, a type of cell death [10]. In addition, previous research has shown that some fatty acid-derived lipids, such as the prostaglandin E_2_ (PGE_2_), regulates NLRP3 and the maturation of IL-1β. PGE_2_ can be associated with inhibition of the NLRP3 activation in human macrophages through the EP4 receptor and by the EP2 receptor in murine macrophages, decreasing IL-1β maturation [11,12]. NLRP3 activation driven by damage-associated molecular patterns is also associated with the production and release of several lipidic mediators, such as eicosanoids derived from arachidonic acids and ceramides, which cause cellular and systemic damages to the organism [13]. Eicosanoids, such as prostaglandins and leukotrienes, are lipid mediators that play a crucial role in initiating the acute inflammatory response [14]. Systemic activation of inflammasomes leads to the production of large amounts of eicosanoids in several minutes, contributing to rapid initiation of inflammation characterized by increased vascular permeability, culminating in pathological inflammatory effects [15]. 

Inflammasome activation is vital for the control of infections and the regulation of metabolic processes and immune responses [16]. However, altered functions of these platforms are implicated in the pathogenesis of several human diseases. Therefore, investigations that highlight novel signaling components that regulate inflammasome activation are crucial to prevent or treat human infections/inflammatory diseases. Considering that AnxA1 is a potent anti-inflammatory protein that down-regulates proinflammatory mediator release, phospholipase A_2_ and, consequently, the critical cascade pathways of eicosanoid production such as that of cyclooxygenase 2 (COX-2) [17,18], this study evaluated its role in regulating the NLRP3 inflammasome and lipid release by macrophages. 

## 2. Materials and Methods

### 2.1. Animals

Male C57BL/6 wild-type (WT) and AnxA1-null (AnxA1^-/-^) mice aged 7–8 weeks and weighing 20–25 g were kept in cages (*n* = 4) in a temperature-controlled environment (22–25 °C) with a 12-h light-dark cycle. They received water and food ad libitum. All animal procedures were approved by the Ethics Committee in Animal Experimentation of the Federal University of São Paulo-UNIFESP (CEUA agreement number: N° 6493130318) and by the Internal Biosafety Commission (CIBio). 

### 2.2. Cell Culture and Treatments 

Lipopolysaccharide (LPS), nigericin, and ATP were obtained from InvivoGen (San Diego, CA, USA). LPS and ATP were reconstituted in endotoxin-free water and nigericin in 100% ethanol. The stock solutions were diluted in endotoxin-free water to prepare intermediate concentration solutions, stored at −20 °C.

WT and AnxA1^-/-^ peritoneal macrophages were obtained by the intraperitoneal injection of a 1.5% starch solution (Sigma Aldrich, St. Louis, MO, USA) in sterile PBS, and after four days, cells were collected by peritoneal wash. Differential cell counts were made on Diff–Quick-stained cell smears prepared by cytocentrifugation. The macrophage population obtained was more than 85% pure and at least 90% viable, as examined by trypan blue exclusion. Additionally, macrophage morphology was confirmed by ultrastructural analysis using transmission electron microscopy. Peritoneal cells (1 × 10^6^ cells/well) were cultured in Opti-MEM (Thermo Fisher Scientific, Waltham, MA, USA) overnight at 37 °C under an atmosphere of 5% CO_2_. Experiments were performed in triplicate in 24-well plates. WT and AnxA1^-/-^ cells were primed with LPS (500 ng/mL for 3 h) followed by stimulation with nigericin (10 μM for 1 h) or ATP (5 mM, 30 min) to activate the NLRP3 inflammasome. 

### 2.3. Analysis of Cell Viability and IL-1β Release

Cell viability was determined by a 3-(4,5-dimethylthiazol-2-yl)-2,5-diphenyltetrazolium bromide (MTT) assay. After the treatment process, the supernatants were discarded, and the RPMI medium (Invitrogen, Gibco, Portland, OR, USA) with 10% MTT solution (5 mg/mL) was added to the cells. After incubating the cells for 4 h at 37 °C under a 5% CO_2_ atmosphere, 300 µL of dimethyl sulfoxide (DMSO; Sigma Aldrich, St. Louis, MO, USA) was added to each well (24-well plate), and 100 µL triplicates of the same sample were transferred to a 96-well plate. The spectrophotometric absorbance values at 490 nm were determined. The percentage of viable cells was calculated by optical density normalization for LPS-stimulated cells only.

IL-1β levels were tested in culture supernatants by an enzyme-linked immunosorbent assay (ELISA) using a commercially available immunoassay kit (BioLegend, San Diego, CA, USA), according to the manufacturer’s instructions. All experiments were conducted in duplicate, and the data were expressed as the mean ± standard error of the mean (SEM) of protein (pg/mL).

### 2.4. Western Blot Analysis

After nigericin and ATP stimulation, the supernatant was removed, and cells were washed three times with sterile PBS, and to each well, 50 µL of lysis buffer was added for cell lysis and protein extraction. Equal amounts of supernatants and cell extracts were loaded onto a 15% sodium dodecyl sulphate-polyacrylamide gel with appropriate molecular weight markers (Bio-Rad Life Science, Hercules, CA, USA) for electrophoresis and transferred to ECL Hybond nitrocellulose membranes. Reversible protein staining of the membranes with 0.1% Ponceau-S in 5% acetic acid (Santa Cruz Biotechnology, Dallas, TX, USA) was used to verify protein transfer. Membranes were incubated 30 min in 5% milk in Tris-buffered saline (TBS) prior to incubation with the antibodies. Primary antibodies were rabbit polyclonal anti-AnxA1 (Invitrogen-Thermo Fisher Scientific, Waltham, MA, USA; 1:1000), goat polyclonal anti-IL-1β (R&E Systems, MN, USA; 1:500), mouse monoclonal anti-caspase-1 (Santa Cruz Biotechnology Dallas, TX, USA; 1:200), and polyclonal rabbit anti-β-actin (Cell Signaling Technology, Beverly, MA, USA; 1:1000), all diluted in TBS. The membranes were then incubated with the appropriate peroxidase-conjugated secondary antibodies (Millipore Corporation, Burlington, MA, USA; 1:2500). Finally, membranes were washed for 15 min with TBS, and immunoreactive proteins were detected (Clarity™ Western ECL Substrate; Bio-Rad, Hercules, CA, USA) using a GeneGnome5 chemiluminescence detection system (SynGene, Cambridge, UK).

### 2.5. Ultrastructural Immunocytochemical Analysis 

WT and AnxA1^-/-^ nigericin-stimulated macrophages were fixed in 4% paraformaldehyde and 0.5% glutaraldehyde, 0.1% sodium cacodylate buffer (pH 7.4) for 24 h at 4 °C. Samples were washed in sodium cacodylate, dehydrated through a graded methanol series, and embedded in LR Gold (Sigma Aldrich Corp., St. Louis, MO, USA).

To detect AnxA1 and NLRP3, ultrathin macrophage sections (~90 nm) were submitted for immunocytochemistry, as previously described [19]. To detect the proteins, the sheep polyclonal antibody anti-AnxA1 (1:200) and rabbit polyclonal antibody anti-NLRP3 (1:200; Cusabio, Houston, TX, USA), following a donkey anti-sheep IgG and goat anti-rabbit IgG antibody (1:50) conjugated to 10-nm and 20-nm colloidal gold (British Biocell, Cardiff, UK), respectively, were used. Ultrathin sections were stained with uranyl acetate and lead citrate and examined using a ZEISS EM900 electron microscope (Carl Zeiss, Jena, Germany). Randomly photographed sections of macrophages were analyzed using Axiovision software. The density of immunogold (number of gold particles/µm^2^) was calculated and reported as the mean ± SEM of 20–40 cells per experimental condition.

### 2.6. Lipidomic Analysis

After treatments, WT and AnxA1^-/-^ cell supernatants (1 × 10^6^ cells/well) were collected and stored at −80 °C until sample processing. For lipid extraction, each sample was randomized and resuspended in 1 mL of 1:2 CHCl_3_: MeOH solution (Sigma Aldrich, Basel, Switzerland), followed by the addition of 0.33 mL CHCl_3_ and 0.33 mL deionized water. The solution was stirred for 5 min, then centrifuged at 13,000 rpm for 5 min. Derived-organic fractions with lipids were collected from the bottom layer of the tubes and transferred to 1.5-mL glass tubes. These fractions were dried in a SpeedVac Savant SPD131DDA concentrator (Thermo Scientific) for 30 min at 30 °C and stored at −80 °C. Mass spectrometric analysis was performed in an ultra-high-performance liquid chromatography (UHPLC) Agilent 1290 Infinity system (Agilent, Santa Clara, CA, USA) and chromatographic elution in a Kinetex C18 column (4.6 mm × 50 mm × 2.6 μm) (Phenomenex, Torrance, CA, USA). All samples were randomized before injection and analyzed by the positive and negative mode in a hybrid mass spectrometer with QTOF 6550 mass analyzer (Agilent, Santa Clara, CA, USA). The mass spectra were acquired in centroid mode, and the mass range used for the acquisition was 50–1700 Da. The raw data were converted by the MassHunter Qualitative software (Agilent, Santa Clara, California, USA) and then imported to XCMS online software (Version 3.7.1, Scripps Center for Metabolomics, La Jolla, CA, USA). For the final statistical analysis, the Metaboanalyst 3.0 platform was used (McGill University, Montreal, Quebec, Canada), as well as the potential lipid biomarkers annotation by the measurement of their exact mass, retention time, and elution profile in METLIN (Scripps Center for Metabolomics, La Jolla, CA, USA), Human Metabolome Database (HMDB) (http://www.hmdb.ca/metabolites), and Lipid Maps databases (http://www.lipidmaps.org/).

### 2.7. Statistical Analyses

The data were analyzed using GraphPad Prism 5.0 software. Results were confirmed to follow a normal distribution using the Kolmogorov–Smirnov test of normality with Dallal–Wilkinson–Lillie for corrected *P*-value. Data that passed the normality assumption were analyzed using analysis of variance (ANOVA) with a Bonferroni post hoc test. Data failing the normality assumption were analyzed using the non-parametric Kruskal–Wallis test followed by Dunn’s post-test, and differences were considered statistically significant at a value of *p* < 0.05.

## 3. Results

### 3.1. The Lack of Endogenous AnxA1 Exacerbates the IL-1β Release and Increases NLRP3 Levels after Inflammasome Activation

**Figure 1:**
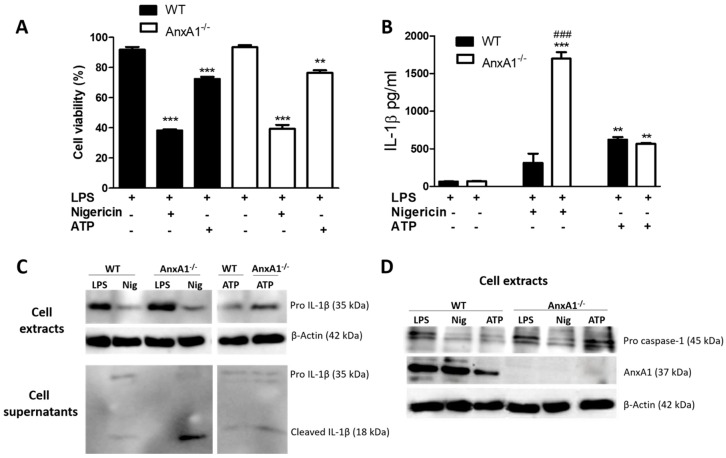
Lack of endogenous Annexin A1 (AnxA1) produced a marked release of IL-1β in macrophages. (**A**) 3-(4,5-dimethylthiazol-2-yl)-2,5-diphenyltetrazolium bromide (MTT) assay. Macrophages of both genotypes showed a marked decrease in cell viability under nigericin and ATP exposure. The percentage of viable cells was calculated by optical density normalization for only lipopolysaccharide (LPS)-stimulated cells. Data are shown as mean ± S.E.M. of cell ratio (%). ** *p* < 0.01; *** *p* < 0.001 vs. LPS-stimulated cells of corresponding genotype (ANOVA, Bonferroni post-test). (**B**,**C**) IL-1β levels. Treatment with nigericin produced a marked release of IL-1β in AnxA1-null (AnxA1^-/-^) macrophages compared with the other groups. Increased levels of pro-IL-1β were detected in the LPS-stimulated wild-type (WT) and AnxA1^-/-^ cell extracts. Values are expressed as mean ± SEM of IL-1β levels (pg/mL). *** *p* < 0.001, ** *p* < 0.01 vs. LPS-stimulated cells of respective genotype; ^###^ *p* < 0.001 vs. WT nigericin-treated cells (ANOVA, Bonferroni post-test). (**D**) Pro caspase 1 and AnxA1 levels in the cell extracts under different experimental conditions. β-actin was used as an endogenous control (representative image of three experiments performed).

First, we verified the endogenous effect of AnxA1 on the activation and regulation of NLRP3 inflammasome in macrophages. As expected, the administration of nigericin caused a significant reduction in cell viability (Figure 1A) without a difference between the two genotypes. Both nigericin and ATP induced IL-1β release by macrophages, which was more pronounced in nigericin-stimulated AnxA1^-/-^ cells than in their respective controls (Figure 1B,C). This latter result was corroborated by the presence of mature IL-1β observed in AnxA1^-/-^ cells (Figure 1C), and pro caspase 1 (Figure 1D) in the same experimental condition. ATP-stimulated WT macrophages presented decreased levels of AnxA1 compared with nigericin-stimulated cells (Figure 1D). In addition, pro caspase 1 levels were similar between only primed and ATP-stimulated AnxA1^-/-^ cells (Figure 1D). 

**Figure 2:**
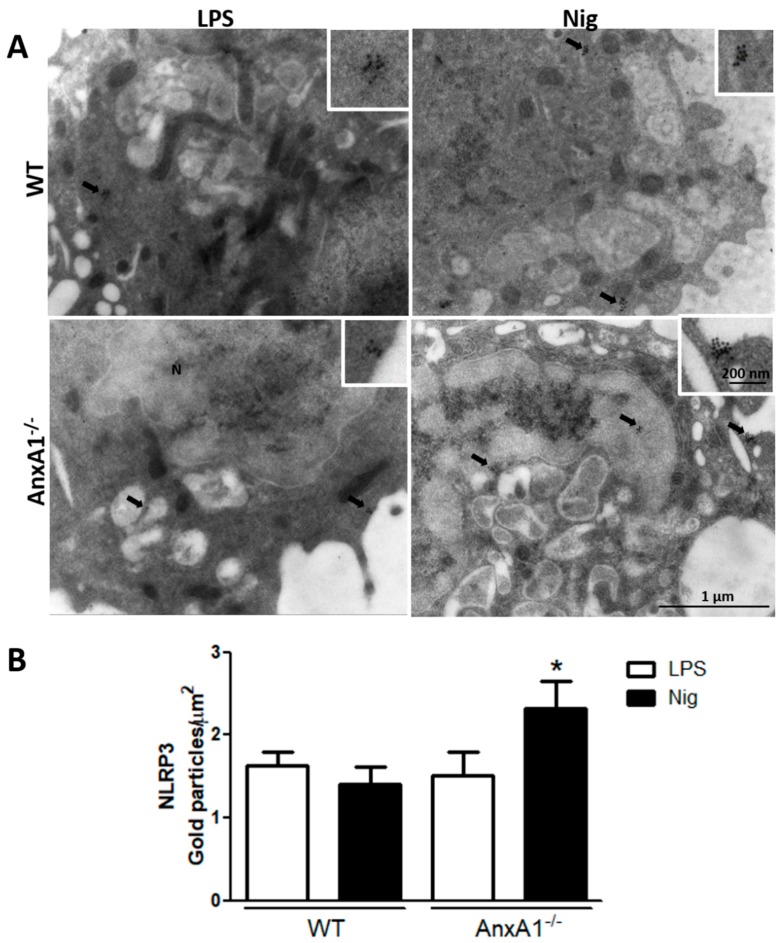
Lack of endogenous AnxA1 increased NLRP3 levels in nigericin-stimulated macrophages. (**A**) NLRP3 expression detected in the cytoplasm (arrows) of cells. Insets: details of cytoplasmic gold labelling of NLRP3. (**B**) Density of NLRP3 immunogold particles in macrophages. Data are mean ± SEM of distinct cells analyzed for each condition. * *p* < 0.05 vs. LPS of corresponding genotype (ANOVA, Bonferroni post-test).

**Figure 3:**
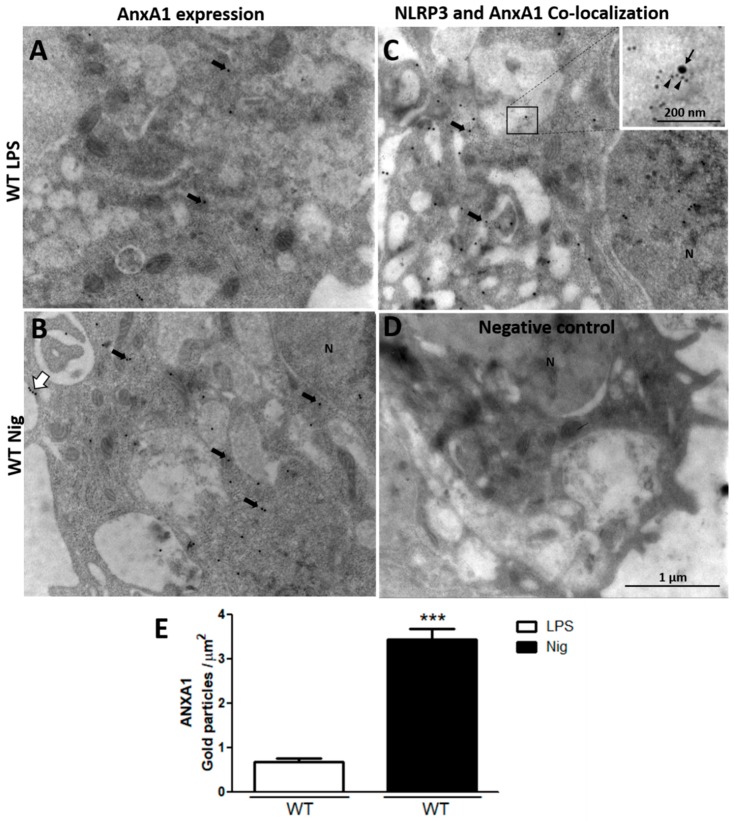
Nigericin-stimulated WT macrophages increase AnxA1 endogenous levels. (**A**,**B**) AnxA1 expression detected in the cytoplasm (arrows) and plasma membrane (white arrow) of cells. (**C**) Points of co-localization of AnxA1 (arrowheads) and NLRP3 (arrow) were detected in the cytoplasm of nigericin-stimulated cells. (**D**) Negative control. (**E**) Density of AnxA1 immunogold particles in macrophages. Data are mean ± SEM of distinct cells analyzed for each condition (*t*-test). *** *p* < 0.001 vs. LPS.

After detecting that nigericin-stimulated AnxA1^-/-^ cells exhibited exacerbated IL-1β production, the AnxA1 and NLRP3 levels were analyzed using ultrastructural immunocytochemistry. The lack of endogenous AnxA1 was associated with increased levels of NLRP3 in nigericin-stimulated macrophages compared with the primed AnxA1^-/-^ cells and WT cells (Figure 2). In contrast, WT nigericin-stimulated macrophages showed a marked increase of AnxA1 levels compared with the only primed cells (LPS) (Figure 3A,B,E). In addition, points of co-localization between AnxA1 and NLRP3 were detected in the cytoplasm of nigericin-stimulated cells (Figure 3C). No immunogold labelling was detected in the negative control of the reaction (Figure 3D).

### 3.2. NLRP3 Activation Induces Different Lipid Release by WT and AnxA1^-/-^ Macrophages

**Figure 4:**
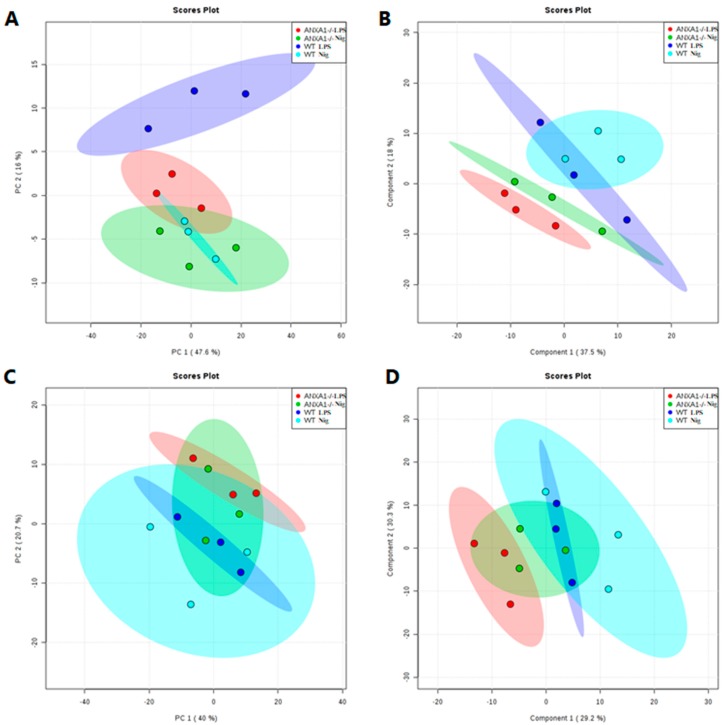
Principal component analysis (PCA) and partial least squares discriminant analysis (PLS-DA) score plots of the lipid fraction. Each spot represents one supernatant sample from control (LPS-stimulated cells - WT: dark blue; AnxA1^-/-^: red) and nigericin-stimulated cells (WT: light blue; AnxA1^-/-^: green). The ellipses show the differences and similarities between groups. (**A**,**C**), PCA in positive and negative ion mode, respectively. (**B**,**D**), PLS-DA in positive and negative ion mode, respectively.

To investigate whether the lack of endogenous AnxA1 also alters lipidomic profiling of macrophages under NLRP3 activation, lipidomic analysis of cell supernatants was performed. Figure 4 shows the score plots of principal component analysis (PCA) and partial least squares discriminant analysis (PLS-DA) obtained in positive and negative ion modes. As expected, the lipid profiles from the WT and AnxA1^-/-^ control groups (LPS-stimulated cells) were different from each other, as shown by the separated dark blue and red ellipses in PCA and PLS-DA, either in the positive and negative ion modes. In the positive mode of the PCA analysis, the first two components of the score plot described 63.6% of explained variation in which the WT LPS separation from the WT treated with nigericin is more pronounced (Figure 4A) in PCA, whereas PLS-DA shows overlapped ellipses (Figure 4B). The negative mode in PCA and PLS-DA analysis showed similar results between groups, as evidenced by the overlapping of ellipses (Figure 4C,D).

**Figure 5:**
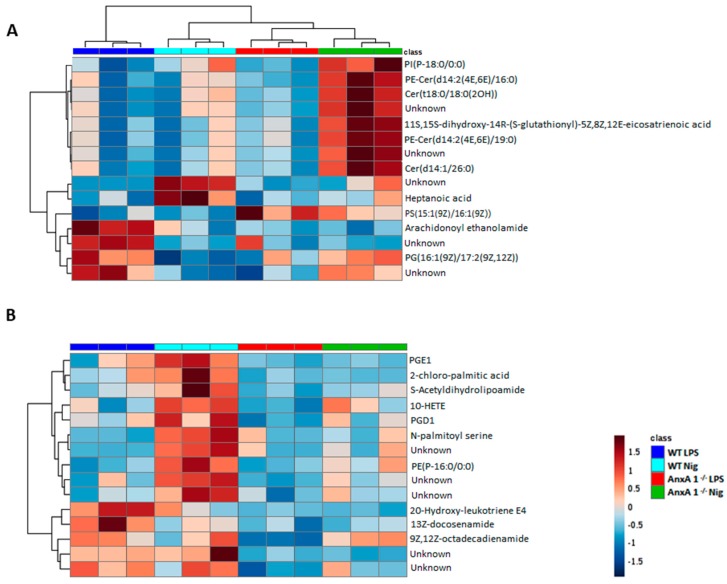
Lipidomic analysis of WT and AnxA1^-/-^ macrophage supernatants. Heatmaps and dendrograms show the hierarchical clustering of potential lipid biomarkers of the control (WT: dark blue; AnxA1^-/-^: red) and nigericin-stimulated cells (WT: light blue; AnxA1^-/-^: green). (**A**) Positive mode. (**B**) Negative mode. Lipidomic analysis demonstrated a completely different lipid profile between WT and AnxA1^-/-^ supernatant cells. In WT cells, nigericin induced a pronounced release of eicosanoids and prostaglandins, while AnxA1^-/-^ cells showed precursors of prostaglandin and some ceramides. The right bar in Figure B represents the blue–red code (−1.5 to 1.5) of the lipid concentrations. Unknown: noncharacterized lipids.

Heatmaps and dendrograms highlight the normalized concentrations of different lipids in the supernatants of WT and AnxA1^-/-^ macrophages. In the positive mode (Figure 5A), the majority of potential lipid biomarkers were produced by nigericin-stimulated AnxA1^-/-^ cells, if compared with the other experimental conditions. These cells showed increased production of sphingolipids, especially ceramides Cer(t18:0/18:O(2OH)), Cer(d14:1/26:0), PE-Cer(d14:2(4E,6E)/16:0), and PE-Cer(d14:2(4E,6E)/19:0) (Figure 5A). In addition, supernatants from nigericin-stimulated AnxA1^-/-^ exhibited a higher concentration of the eicosanoid 11S-15S-dihydroxy-14R-(S-glutathionyl)-5Z,8Z,12E-eicosatrienoic acid and the neutral lipid PI(P-18:0/00). In contrast, heptanoic acid was identified as a potential lipid biomarker in the supernatants of nigericin-stimulated WT cells, with a higher concentration than control WT cells (LPS), while arachidonoyl ethanolamide and the plasma membrane compound phosphatidylglycerol PG (16:1(9Z)/17:2(9Z,12Z)) were lower. PS (15:1(9Z)/16:1(9Z)) is a phosphatidylserine which only appeared in the supernatants of AnxA1^-/-^ control cells and those treated with nigericin (Figure 5A). 

Figure 5B shows the potential lipid biomarkers in the negative mode. In the supernatants of nigericin-stimulated WT cells, the more concentrated lipids were associated with the arachidonic acid metabolism, such as the 10-hydroxyeicosatetraenoic acid (10-HETE), prostaglandins D1 and E1 (PGD1, PGE1), as well as palmitic acid, S-acetyldihydrolipoamide, N-palmitoyl serine, and PE(P-16:0/0:0). Curiously, there are different lipid profiles in the supernatants of WT and AnxA1^-/-^ control cells, as characterized by a higher concentration of the eicosanoid 20-hydroxy-leukotriene E4 and fatty acid amides (13Z-docosenamide and 9,12Z-octadecadienamide) in the WT cells. 

## 4. Discussion

Monocytes and macrophages express NOD-like receptors, which are very important for the immune response during inflammation [20]. By NLRP3 inflammasome activation in these cells, pro-IL-1β and pro-IL-18 are cleaved and released, increasing the pro-inflammatory response [21]. Once macrophages are activated by the NLRP3 inflammasome agonists, such as nigericin and ATP, there is an IL-1β production and release, and the K^+^ concentration is reduced [22]. IL-1β is a proinflammatory cytokine, and once it is released by macrophages, IL-6, TNF, nitric oxide, and prostaglandin E_2_ (PGE_2_) are produced [23]. Our data provide previously unknown details regarding the interplay between AnxA1 and NLRP3-derived IL-1β in macrophages and its relationship with lipid-mediator release. 

Under normal conditions, the AnxA1 protein is present in high levels in the cytoplasm of human and rodent leukocytes, such as neutrophils, monocytes, and macrophages, and once these cells are activated, the AnxA1 moves to the cell membrane to be released and act as an autocrine or paracrine mediator [24,25]. The ATP-binding cassette transporter is responsible for AnxA1 secretion in macrophages [26], and under conditions of cellular stress, AnxA1 is rapidly released [27]. 

Our results show that the lack of AnxA1 exacerbates the IL-1β production after NLRP3 activation. These findings were supported by increased levels of NLRP3 in AnxA1^-/-^ macrophages, as observed by ultrastructural immunocytochemistry. Considering that peritoneal AnxA1^-/-^ macrophages presented increased levels of TLR4 [6], the lack of AnxA1 could favor LPS “over-priming” and consequent increase in NLRP3 inflammasome-derived IL-1β secretion. In addition, nigericin stimulation increased endogenous AnxA1 that presents points of co-localization with NLRP3 in WT macrophages, supporting a role of AnxA1 in the activation and regulation of the NLRP3 inflammasome. Regarding ATP stimulation, western blotting detected more decreased levels of AnxA1 in WT macrophages than in the priming LPS and nigericin-stimulated cells. In fact, activation of the P2 × 7 receptor by extracellular ATP in macrophages has been widely studied as a trigger of the NLRP3 inflammasome and is associated with the release of AnxA1 [7]. However, this study also demonstrated that nigericin did not induce AnxA1 release, suggesting pathways within P2 × 7R signaling in addition to K+ efflux in macrophages for the release of this protein. 

Despite our findings, recent analyses show that bone-marrow-derived AnxA1^-/-^ macrophages produced significantly lower secretion of IL-1β when activated with monosodium urate (MSU) crystals and ATP, but not NLRC4 or AIM2 activators (*Legionella pneumophila* or poly(dA:dT)) [28]. Notably, during the setting of MSU crystal-induced inflammation, the peak of the neutrophil influx was greater, and the resolution was slower in AnxA1^-/-^ mice than in WT animals [29]. These findings are consistent with many studies that have shown an exacerbated inflammatory response in AnxA1^-/-^ mice characterized by a marked leukocyte influx and production of proinflammatory mediators, such as IL-1β and IL-6 [4,6,30,31,32]. Additionally, the administration of the AnxA1 peptide (Ac2–26) 1 h before and 12 h after challenge with MSU crystals induced decreased levels of IL-1β in periarticular tissue, showing the important anti-inflammatory and proresolving activity of this protein on the course of MSU crystal-induced inflammation in mice [29]. Thus, the opposite effects described for the role of AnxA1 on NLRP3 inflammasome-derived IL-1β secretion could be a result of testing different macrophage populations, bone-marrow versus peritoneal-derived, and different strains, BALB/c versus C57BL/6, an important factor in the mouse immunology response [33,34]. 

The NLRP3 inflammasome is a cytosolic platform formed by a multi-protein complex containing a nucleotide-binding oligomerization domain-like receptor and the adaptor apoptosis-associated spec-like protein (ASC) containing an amino-terminal caspase-recruitment domain (CARD) [9]. The interaction of the NLRP3 with ASC promotes the recruitment of the procaspase-1 and its autoproteolysis, driving IL-1β and IL-18 maturation, membrane pore formation by the gasdermin D action, and then cytokine secretion and pyroptosis [35,36]. Besides the cleavage and release of the IL-1β, the activation of the NLRP3 inflammasome is directly related to the production of lipid mediators, including eicosanoids and ceramides. These lipids can be involved in metabolic and immunological pathways, and the deregulation of NLRP3 activation causes an increase of the lipidic mediators released after pyroptosis, and it is possible to conduct metabolic damage on a systemic level [13]. 

The current lipidomics approach is a great tool to understand biological systems and many diseases. The major lipid classes can be categorized as fatty acyls, glycerolipids, glycerophospholipids, sphingolipids, sterol lipids, prenol lipids, saccharolipids, and polyketides [37]. In addition, cells can synthesize lipids, such as the eicosanoids, which are derived by the arachidonic acid oxidation and represented by prostaglandins, leukotrienes, thromboxanes, lipoxins, and epoxyeicosatrienoic acids [38]. By providing the exact mass, retention time, and elution profile, our study presents new data about the lipidomic profile of the supernatant of macrophages after NLRP3 activation by nigericin. 

In our study, we showed the ceramides PE-Cer(d14:2(4E,6E)/16:0), Cer(t18:0/18:O(2OH)), PE-Cer(d14:2(4E,6E)/19:0), and Cer(d14:1/26:0) as potential lipid biomarkers released by AnxA1^-/-^ macrophages after nigericin stimulation. Ceramides are bioactive sphingolipids present in the plasma membrane and they mediate cell signaling, with a close relationship to many pathophysiological processes associated with inflammation [39]. Previous studies showed that the exposure of macrophages to ceramides causes activation of caspase-1, and this effect is prevented by the absence of NLRP3 [40]. Ceramides have also been related to the activation of TLR4, augmenting LPS-induced pro-inflammatory response [41]. In this regard, it is reasonable to infer that increased levels of TLR4 in AnxA1^-/-^ macrophages [6] contribute to the more pronounced ceramide concentration in their supernatants and also in NLRP3 activation. 

Supernatants from nigericin-stimulated AnxA1^-/-^ macrophages also exhibited a higher concentration of the 11S-15S-dihydroxy-14R-(S-glutathionyl)-5Z,8Z,12E-eicosatrienoic acid, a type of eicosanoid, and PI(P-18:0/0:0). Some studies have shown that 12-hydroxyeicosatrienoic acid (12-HETE) plays an important role as a paracrine mediator of inflammation, as well as in the regulation of neutrophil infiltration in damaged tissue [42,43]. PI(P-18:0/0:0) is an important phosphatidylinositol in cell membranes and for metabolic processes, such as being the primary source of arachidonic acid metabolism for eicosanoid synthesis and intracellular signals in animal tissues [44]. The lack of AnxA1 in macrophages increased the arachidonic acid metabolism and eicosanoid production, as evidenced by the high concentration of 11S-15S-dihydroxy-14R-(S-glutathionyl)-5Z,8Z,12E-eicosatrienoic acid and PI(P-18:0/0:0) after NLRP3 activation and pyroptosis induction. The 11S-15S-dihydroxy-14R-(S-glutathionyl)-5Z,8Z,12E-eicosatrienoic acid is present in leukocytes and red blood cells, and earlier studies have demonstrated that this fatty acid can be converted by arachidonic acid by the lipoxygenase pathway [45,46], which is basically synthesized during the inflammatory process [47]. 

In the supernatants of nigericin-stimulated WT macrophages, more concentrated lipids are also associated with the arachidonic acid metabolism, such as the 10-HETE, PGD1, PGE1, as well as palmitic acid, S-acetyldihydrolipoamide, N-palmitoyl serine, PE(P-16:0/0:0), and heptanoic acid, indicating a completely different lipid profile of AnxA1^-/-^ supernatants; 10-HETE is a hydroxyeicosatrienoic acid with proinflammatory action, increasing TNF-α and IL-6 production in macrophages [48]. In contrast, PGE1 and PGD1 are anti-inflammatory lipid mediators that inhibit leukocyte migration and adhesion and mast cell activation [49,50,51]. Palmitic acid (PA), also called hexadecanoic acid, is one of the most common saturated fatty acids in animals. PA acts as a lipid mediator in inflammation and its derived-metabolic products accumulate in the endoplasmic reticulum (ER) and increase reactive oxygen species (ROS) generation, leading to cell death [52]. Additionally, the inflammatory response caused by ER stress and ROS generation from high concentrations of PA drives NF-κB and NLRP3 activation and, consequently, proinflammatory cytokine release by monocytes/macrophages [53,54,55,56]. S-Acetyldihydrolipoamide, N-palmitoyl serine and PE(P-16:0/00) (2-Hexadecanoyl-1-(1Z-hexadecenyl)-sn-glycero-3-phosphoethanolamine) are associated with cell metabolism [57], membrane receptor [58], and cell membrane compounds [59], respectively. However, there is little information about heptanoic acid in biological systems. Metabolomic studies have shown a high concentration of heptanoic acid in the faeces of autistic children [60], while in patients with Crohn’s disease, ulcerative colitis, and pouchitis, lower levels of this lipid were found [61]. Altogether, our data show that macrophages can release potential lipid biomarkers after NLRP3 activation that can regulate the inflammatory responses in the damaged tissue.

This study also detected a different lipid profile in supernatants from WT and AnxA1^-/-^ LPS-stimulated macrophages. Higher concentrations of the eicosanoid 20-hydroxy-leukotriene E4 and fatty acid amides (13Z-docosenamide and 9,12Z-octadecadienamide) were found in the WT samples, while PS(15:1(9Z)/16:1(9Z)) was found in AnxA1^-/-^ samples. In addition, 20-hydroxy-leukotriene E4 is an eicosanoid metabolite originating from the lipid oxidation of leukotriene E4 that plays an essential role in cell proliferation, differentiation, and immunoregulation [62]. Moreover, the leukotriene E4 contributes to prolonged intracellular signaling, increasing intracellular Ca2^+^ and ERK phosphorylation [63], confirming signal pathway triggering by LPS on WT macrophages. Biological functions for both fatty acid amides detected in this study still need to be addressed. 

Finally, PS(15:1(9Z)/16:1(9Z)) is a phosphatidylserine (PS) involved in cell signaling, including an important role in cell death, either by apoptosis, necroptosis, or pyroptosis, by its exposure on the outer plasma membrane layer [64]. The detection of this potential lipid biomarker in both AnxA1^-/-^ supernatants (LPS and nigerin) can be related to the release of extracellular vesicles by macrophages. Macrophages can release extracellular vesicles after *Mycobacterium tuberculosis* infection, or spontaneously with a high concentration of phosphatidylserine [65]. 

## 5. Conclusions

The lack of AnxA1 favors LPS “over-priming” and the release of lipid mediators (e.g., ceramides) that produce exacerbated NLRP3 activation under nigericin stimulation. Although more detailed investigations are warranted, this study identifies AnxA1 as a novel signaling component of inflammasome activation and a potential therapeutic target to treat inflammatory diseases.
